# Selective Inactivation Strategies for Vegetable Raw Materials: Regulating Microbial Communities to Ensure the Safety and Quality of Fermented Vegetables

**DOI:** 10.3390/foods14193291

**Published:** 2025-09-23

**Authors:** Lin Zhu, Mengke Cheng, Cuicui Xu, Rong Wang, Meng Zhang, Yufei Tao, Shanshan Qi, Wei Wei

**Affiliations:** 1 School of Agricultural Engineering, Jiangsu University, Zhenjiang 212013, China; zhulin19820402@ujs.edu.cn (L.Z.); 2212316009@stmail.ujs.edu.cn (M.Z.); 2212416006@stmail.ujs.edu.cn (Y.T.); qishanshan1986120@163.com (S.Q.); 2 School of Food and Biological Engineering, Jiangsu University, Zhenjiang 212013, China; 2212318054@stmail.ujs.edu.cn (M.C.); 1000004488@ujs.edu.cn (C.X.); 2222418036@stmail.ujs.edu.cn (R.W.)

**Keywords:** fermented vegetables, selective inactivation, cold plasma, electromagnetic wave, natural essential oils, LAB metabolites, synergistic strategies

## Abstract

Fermented vegetables, which are valued for their distinctive organoleptic properties and nutritional profile, are susceptible to quality deterioration during processing and storage because microorganisms inhabit vegetable raw materials. The metabolic processes of these microorganisms may induce texture degradation, chromatic alterations, flavor diminution, and spoilage. Conventional inactivation methods employing thermal sterilization or chemical preservatives achieve microbial control through nonselective inactivation, inevitably compromising the regional sensory characteristics conferred by indigenous fermentative microbiota. Recent advances in existing antimicrobial technologies offer promising alternatives for selective microbial management in fermented vegetable matrices. Existing modalities, including cold plasma, electromagnetic wave-based inactivation (e.g., photodynamic inactivation, pulsed light, catalytic infrared radiation, microwave, and radio frequency), natural essential oils, and lactic acid bacterial metabolites, demonstrate targeted pathogen inactivation while maintaining beneficial microbial consortia essential for quality preservation when properly optimized. This paper explores the applications, mechanisms, and targeted microbes of these technologies in fermented vegetable ingredients, aiming to provide a robust theoretical and practical framework for the use of selective inactivation strategies to manage the fermentation process. By assessing their impact on the initial microbial community, this review aims to guide the development of methods that ensure product safety while safeguarding the characteristic flavor and quality of fermented vegetables.

## 1. Introduction

Fermented vegetables, as traditional food processing systems driven by microbial community succession, not only impart unique flavor, textures, and sensory enjoyment to the product but also significantly increase its nutritional value and potential health benefits [1,2,3]. However, during the processing and storage of fermented vegetables, the microorganisms on the surface of vegetable raw materials are easily disturbed by the surrounding environment, which makes fermented vegetables at risk of softening in texture, losing color, losing flavor and even deteriorating [4,5]. Of particular concern is the overgrowth or abnormal activity of specific microbial groups within the fermentation system, such as certain spoilage bacteria/pathogenic bacteria, yeasts, and molds, which have become core challenges that threaten product storage stability and edible safety [6,7,8].

Traditionally, mainstream antimicrobial and preservative strategies for vegetable ingredients have relied on physical thermal sterilization techniques or chemical means based on synthetic preservative additives [9]. Pasteurization, a sterilization method with a temperature below 100 °C, can preserve the nutrients of fermented vegetables to a certain extent and their sensory qualities such as texture and color. Essentially, it inactivates the microbial community without discrimination [10,11]. Similarly, chemical preservatives like sodium benzoate and potassium sorbate are widely used for their efficiency and convenience. However, when it comes to inhibiting microorganisms that are harmful to the fermentation process, most of the time, these chemical preservatives will significantly interfere with or even inhibit the activity of local beneficial microorganisms required during the fermentation process. The local beneficial microorganisms mentioned here mainly refer to lactic acid bacteria. Such interference and inhibition may disrupt the integrity of the fermentation process and may also change the typical flavor characteristics that the final product should originally have [12,13]. Therefore, these “one-size-fits-all” preservative and sterilization methods, while addressing safety issues, often unavoidably sacrifice the core value of the product shaped by a specific microbial community structure—the regional characteristic flavor and quality [8,14].

However, the research paradigm has shifted in the past decade, gradually transitioning from “comprehensive eradication” to “precision regulation” [15]. Unprecedented developments in the fields of physical processing, chemical additives, and biological methods based on LAB metabolites in food preservation and antimicrobial strategies have given rise to many existing inactivation technologies [16,17,18,19]. Crucially, some of these technologies can not only effectively target and inhibit foodborne pathogens but also actively reshape the indigenous microbial community structure of foods (especially fermented foods) through their selective effects [20,21]. This alteration in microbial community structure has profound, and sometimes pleasantly surprising, impacts on the final product′s quality and flavor characteristics [22]. For example, recent research findings indicate that cold plasma, under specific parameters, can achieve selective inactivation of the indigenous microorganisms of vegetable ingredients. By achieving this selective inhibitory effect, the nitrite metabolism pathway during the subsequent fermentation process of fermented vegetables can be effectively regulated, and the overall quality and complexity of the flavor of the vegetables can be significantly enhanced [23]. This study not only explores the “added value” of nonthermal physical inactivation technologies in detail but also provides a paradigm for the innovative application of similar technologies. Therefore, the core of this review is to systematically summarize many existing preservation and antimicrobial technologies currently applied in the preservation of fermented vegetable raw materials and to focus on introducing those with selective inactivation effects. To ensure a comprehensive and unbiased literature collection, a systematic search strategy was implemented. Relevant scientific publications were retrieved from electronic databases including Web of Science, Scopus and PubMed, covering the period from January 2000 to December 2024. The search utilized a combination of keywords such as selective inactivation, inactivation, bacteriostasis, antimicrobial treatment, cold plasma, electromagnetic wave, essential oils, LAB metabolites, fermented vegetables, microbial community, and food safety. The inclusion criteria prioritized peer-reviewed original research articles, reviews, and meta-analyses published in English, with a focus on studies demonstrating antimicrobial efficacy and mechanisms applicable to vegetable matrices. This methodological approach ensured the inclusion of high-quality, relevant literature for a critical and frontier analysis. This article aims to emphasize an analysis of their “selective inactivation” mechanisms and targeted microbial group characteristics and elucidate how different technologies shape the initial microbial community in fermented vegetable fermentation systems, providing a theoretical and practical framework for employing selective inactivation to actively regulate fermentation processes—ensuring safety while precisely optimizing or even innovating product quality and flavor profiles.

## 2. Cold Plasma

**Figure 1:**
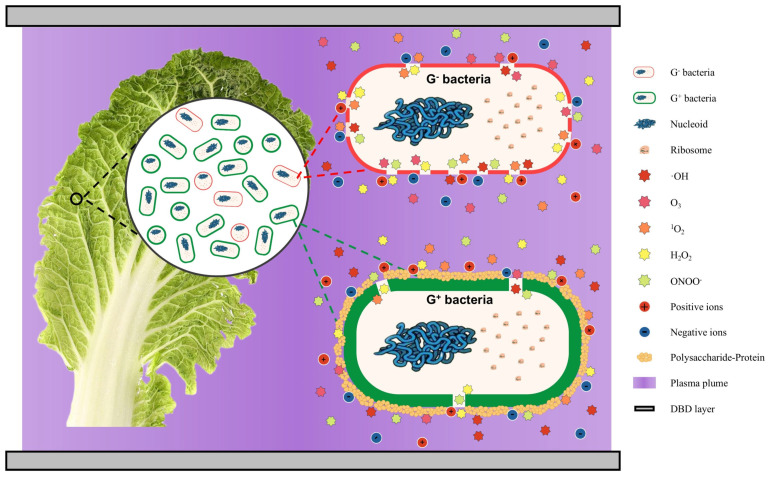
Selective inactivation of Gram-positive and Gram-negative bacteria inhabiting fermented vegetable raw materials via dielectric barrier discharge cold plasma based on RONS and charged particles.

Cold plasma (CP), a novel physical inactivation method, derives its antimicrobial effect from the multimodal synergistic action of reactive oxygen/nitrogen species (RONS, including ·OH, O_3_, ^1^O_2_, H_2_O_2_, ONOO^−^, etc.), charged particles (electron density of 10^14^–10^16^ m^−3^), and ultraviolet photons generated during the plasma phase transition process [24,25,26]. Through excitation methods such as dielectric barrier discharge (DBD) or jet discharge (ionization threshold >10 eV), high-energy electrons (3–5 eV) formed by the dissociation of neutral gases can trigger different inactivation mechanisms for targeted microorganisms [27,28]. Notably, CP selectively sterilizes microbial groups [29,30]. This selectivity primarily arises from differences in the chemical sensitivity of microbial cell wall/membrane structures, with the outer membrane lipopolysaccharide layer of Gram-negative bacteria exhibiting greater permeability to RONS than the thick peptidoglycan layer of Gram-positive bacteria (Figure 1). Additionally, heterogeneity in antioxidant enzyme systems, where microbial strains with strong antioxidant defence capabilities, such as catalase (CAT) and superoxide dismutase (SOD), present significantly higher survival rates (*p* < 0.05), also contributes. Finally, the protective effect of the biofilm matrix, where microbial-secreted polysaccharide-protein complexes can intercept more than 60% of charged particle attacks, plays a role [31] (Figure 1). This selectivity provides a theoretical basis for the targeted removal of pathogens/spoilage bacteria in fermented vegetable ingredients, enabling the inhibition of *Escherichia coli*, *Salmonella*, and other foodborne pathogens while preserving the activity of fermentation-functional microorganisms such as LABs.

### 2.1. Cold Plasma Inactivation on Vegetable Ingredient Surfaces

The selective inactivation effect of cold plasma on vegetable ingredient surfaces is influenced by the morphological differences in the ingredients. Root and stem vegetables with smooth surfaces lack physical shelters for microorganisms, facilitating the antimicrobial action of cold plasma. In contrast, leafy vegetables (such as cabbage) with complex folds on their surfaces can form microbial havens, potentially hindering plasma penetration. Therefore, specific parameter optimization studies are required for different ingredients.

Wei et al. [32] used radish (a typical root vegetable) as the subject and applied dielectric barrier discharge (DBD) cold plasma treatment (40 kV, 60 s), which not only achieved inactivation but also demonstrated additional benefits in nitrite control. The treatment reduced the abundance of potential nitrite-producing Gram-negative bacterial groups (*Pseudomonadaceae* and *Enterobacteriaceae*) on the ingredient surface by 41–74%, while increasing the abundance of acid-producing LABs (mainly *Leuconostocaceae*) to 1.2–6 times that of the control. This microbial community reconfiguration prompted the initiation of the nitrite degradation process one day earlier, ultimately reducing the nitrite peak and cumulative amount to 41.3% and 42.6% of the control group, respectively. For cabbage, a vegetable with a folded epidermis used as an ingredient of fermented vegetable, a suitable cold plasma treatment time of 45 s was determined under fixed voltage conditions of 60 kV on the basis of the inhibition rates of non-LAB groups such as coliforms and yeasts, the survival rate of LAB, and the burning state of the cabbage surface [23]. Under these treatment conditions, the paocai fermented from the treated cabbage presented significantly lower nitrite peaks than those in the control group and the national standards did, with the peak appearing one day earlier, effectively controlling the hazard of nitrite [23]. Further genotypic and phenotypic studies of the strains isolated during this period revealed that cold plasma treatment preferentially eliminated non-LAB groups residing on the cabbage surface, which are capable of producing extracellular nitrite through nitrate dissimilatory reduction (e.g., *Enterobacter asburiae*, *Kluyvera ascorbata*, and *Klebsiella michiganensis*). Moreover, it effectively preserved highly organic acid-producing LABs (e.g., *Lactiplantibacillus pentosus*, *Levilactobacillus brevis*, and *Lactobacillus acetotolerans*). The surviving LAB accelerated the decrease in fermentation broth pH, thereby triggering earlier nitrite degradation [33]. Additionally, fermented vegetables treated with cold plasma not only maintained overall acceptability and color but also experienced significantly enhanced crispness, organic acid content (lactic and acetic acids), and pleasant volatile compound content, improving their taste, flavor, and overall sensory experience [23,32,33].

In summary, cold plasma, when applied under suitable conditions to both root and leafy vegetable ingredients, can prioritize the elimination of Gram-negative bacteria prone to spoilage while preserving Gram-positive fermentation microorganisms—LABs—through their differing plasma sensitivities. Moreover, as targeted Gram-negative bacteria are the primary source of nitrite hazards and acid production by LAB can lead to the chemical conversion of nitrite, controlling nitrite hazards in fermented vegetables has become an “additional function” of cold plasma inactivation technology.

### 2.2. Cold Plasma-Activated Water/Ice for Regulating Surface Microorganisms of Fermented Vegetable Ingredients

Cold plasma-activated water/ice (PAW/PAI) systems construct active water-based inactivation systems through triphasic interface reaction mechanisms [34]. The preparation process includes three modes: gas–liquid interface discharge (power density of 0.5–2 W/cm^3^), direct liquid-phase discharge (gas flow rate of 1–5 L/min), and intraliquid microbubble discharge (bubble diameter < 500 μm). The selective inactivation of the PAW system is achieved through the dynamic coupling of pH and redox potential (ORP) (R^2^ = 0.87): in acidic environments (pH < 3.7), the collapse of the proton motive force induced by the H^+^ gradient preferentially inhibits ATP-dependent microorganisms (such as yeasts); under neutral conditions (pH 5.0–7.0), H_2_O_2_-dominated oxidative stress (ORP > 600 mV) increases the inactivation efficiency of Gram-negative bacteria by 1.5–2.3 log [35]. Studies on the treatment of fermented vegetable ingredients (such as salted mustard) have shown that PAW (H_2_O_2_ 80 μM, pH 3.5) treatment for 5 min can reduce surface spoilage bacteria (*Pseudomonas* species) by 3.2 log CFU/g, while maintaining a LAB survival rate of >85% [36]. This characteristic is crucial for maintaining the fermentation potential of the ingredients, but the treatment intensity needs to be optimized to avoid nonenzymatic oxidation of phenolic substances (increase ≤ 15%) affecting flavor [36].

### 2.3. In-Package Cold Plasma Technology for Inhibiting Secondary Contamination of Fermented Vegetables

In-package cold plasma (ICP) technology achieves in situ generation and targeted delivery of gaseous active components by constructing a closed discharge environment. Through the synergistic effect of modified atmosphere packaging (MAP, O_2_/CO_2_ = 70%:30%) and pulsed discharge (pulse width 100–500 ns), ICP technology can generate gaseous active components (O_3_ 120 ppm, NOx 45 ppm) in situ within a closed environment, extending the shelf life of finished fermented vegetables [37]. Ziuzina et al.’s [38] research results indicate that the selective antimicrobial effect of in-package cold plasma technology is mainly due to the higher mortality rate of Gram-negative bacteria (such as *Escherichia coli*) than that of Gram-positive bacteria (such as *Bacillus*) by 1.8 log (*p* < 0.01), which is caused by outer membrane lipid peroxidation (the increase in the MDA content is 2.8 times greater). Additionally, the electrostatic adsorption effect results in a 67% higher inactivation rate for free-state microorganisms than those within biofilms [36]. Zhang et al. [39] reported that treating kimchi with ICP (10 kV/cm, 3 min) could reduce the risk of *Listeria* contamination to <1 CFU/100 g while maintaining LAB counts >10^7^ CFU/g. Zhou et al. [40], through surface topology effect analysis, reported that the folded structure of vegetable ingredients (Ra > 50 μm) could decrease the plasma penetration efficiency by 40%, necessitating the use of multidirectional electrode designs and dielectric material optimization (PA/LDPE, ε = 3.2) to improve inactivation uniformity.

## 3. Electromagnetic Wave-Based Inactivation

**Figure 2:**
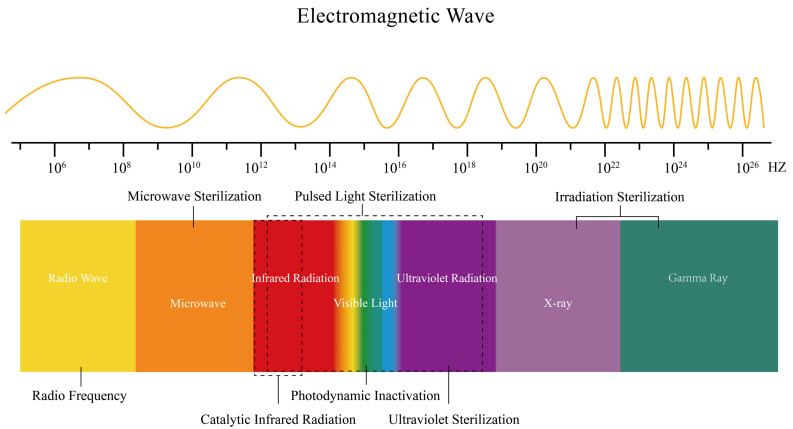
Physical antimicrobial technology involves electromagnetic waves.

Electromagnetic wave spectrum inactivation technology utilizes a specific band energy (10^5^–10^27^ Hz) for microorganism killing on the basis of different mechanisms of action (Figure 2). Among them, certain wavelengths of electromagnetic waves exhibit selective antimicrobial effects, primarily derived from the specific photoabsorption of microbial pigments/photosensitizers, differences in dielectric properties of microbial cell components, and the response threshold differentiation of microbial morphological structures to electromagnetic fields. After sorting through all electromagnetic wave inactivation technologies, five types that can achieve selective microorganism inactivation through parameter adjustment are currently identified.

### 3.1. Photodynamic Inactivation

**Figure 3:**
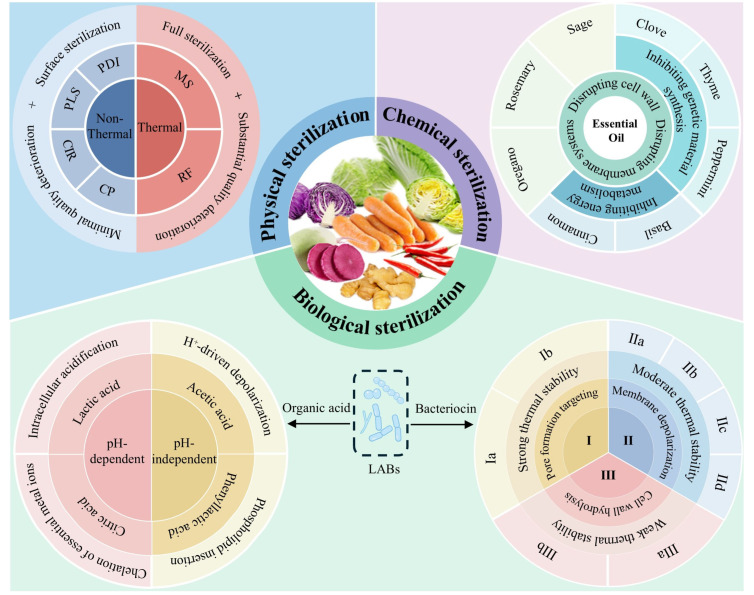
Existing antimicrobial technologies based on physics, chemistry, and biology for selective inactivation of microbial communities in fermented vegetable raw materials. MS: microwave sterilization; RF: radio frequency; CP: cold plasma; CIR: catalytic infrared radiation; PLS: pulsed light sterilization; PDI: photodynamic inactivation; Ia: Lantibiotics; Ib: Cyclic bacteriocins; IIa: Pediocin-like bacteriocins; IIb: Multi-component bacteriocins; IIc: Leaderless circular bacteriocins; IId: Other single-component bacteriocins; IIIa: PG Hydrolase Bacteriocins; IIIb: Large Non-enzymatic Bacteriocins.

**Figure 4:**
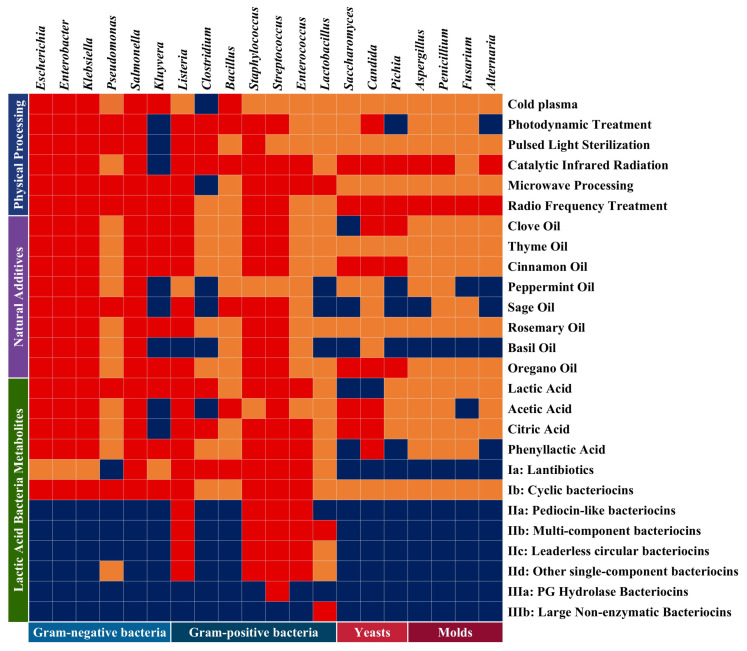
The selective inactivation type of existing antimicrobial technologies discussed herein against common food-associated detrimental and beneficial microorganisms. Red square pattern, preferentially inactivated; orange square pattern, selectively inactivated under modifiable conditions; blue square pattern, recalcitrant to inactivation or not yet characterized.

Photodynamic inactivation (PDI) is a nonthermal antimicrobial technology based on photochemical principles (Figure 3). Through the excitation of photosensitizers by specific spectra (400–700 nm), reactive oxygen species (ROS) are generated to achieve the selective inactivation of microorganisms. Its core mechanism involves a type II photochemical reaction, where in the presence of molecular oxygen, excited-state photosensitizers such as porphyrin compounds transfer energy to oxygen molecules, generating singlet oxygen (^1^O_2_), superoxide anions (O_2_^−^), hydroxyl radicals (·OH) and other highly reactive substances [41]. These ROS induce DNA strand breaks, protein oxidation modifications, and membrane lipid peroxidation, leading to irreversible damage to microorganisms. Compared with traditional thermal and chemical sterilization, PDI technology offers advantages such as nonthermal damage, no residue, and low energy consumption [40]. However, its antimicrobial efficiency is regulated by three factors: photosensitizer characteristics, photosensitizer delivery efficiency, and irradiation parameters. Mushtaq et al. [42] verified that PDI technology has selective antibacterial efficacy against microorganisms. Their study used 405 nm blue light (30 J/cm^2^) combined with 100 μM curcumin, which reduced the colony counts of *Pseudomonas aeruginosa*, methicillin-resistant *Staphylococcus aureus* (MRSA), *Escherichia coli*, and *Candida albicans* by 3.5 log, confirming its broad-spectrum inhibitory ability against multiple foodborne pathogens. Research reports have shown that PDI has a broad inactivation range, encompassing bacteria, yeasts, and molds. By adjusting parameters and intensity, it can inhibit or even kill *Listeria monocytogenes* and *Clostridium sporogenes* (Gram-positive bacteria), *Pseudomonas aeruginosa* and *Escherichia coli* (Gram-negative bacteria), *Candida albicans* (yeast), and *Fusarium graminearum* and *Alternaria alternata* (Figure 4).

Currently, the food industry primarily adopts natural photosensitizers such as curcumin and riboflavin. However, these natural photosensitizers face significant bottlenecks in solid food applications—due to adsorption heterogeneity and insufficient photostability, their antimicrobial efficiency is reduced by 18–35% compared with that of liquid systems, limiting the sterilization of fermented vegetable ingredients. To overcome this limitation, Yang and Shi [43] proposed an innovative photosensitizer-phage synergistic system in which the specific penetration of biofilms by phages significantly increases the bioaccessibility of ROS. Experiments revealed that 50 μM curcumin combined with phage treatment could reduce *Shigella* by 2.77 log, whereas the 100 μM treatment group achieved a 3.64 log inactivation rate against *Salmonella*. This strategy provides a new paradigm for the precise inactivation of solid vegetable ingredients.

### 3.2. Pulsed Light

Pulsed light (PLS) is a well-established nonthermal inactivation technology based on broad-spectrum instantaneous light irradiation [44,45] (Figure 3). Its core mechanism originates from the 100–1100 nm wide-spectrum radiation generated by the instantaneous discharge of a dedicated capacitor [46], which encompasses two characteristic action intervals: the ultraviolet spectrum region (100–400 nm) and the infrared spectrum region (800–1100 nm). Its antimicrobial efficacy stems from the triple synergistic mechanism of UV–infrared composite spectra, including photophysical effects, photothermal effects, and photochemical effects [47]. The photophysical effect mechanism involves mechanical stress effects triggered by instantaneous high-energy photon impacts (>1 J/cm^2^), leading to damage to the cell wall structure and physical rupture of intracellular components [48]. The photothermal effect mechanism is attributed to the infrared band (800–1100 nm), which, through molecular vibration energy level transitions, generates instantaneous high temperatures of 100–150 °C on a picosecond time scale, causing phase transitions in microbial membrane lipid bilayers and destabilization of transmembrane protein conformations [45]. The photochemical effect refers to the formation of cyclobutane pyrimidine dimers between DNA strands induced by the UV-C band (200–280 nm), as well as the excitation of endogenous photosensitizers to produce ROS, resulting in nucleic acid photolysis and enzyme system dysfunction [49].

Owing to the structural differences in microbial cell walls and membranes, the photophysical and photothermal effects of PLSs inevitably differ, ultimately leading to selective inactivation effects on microorganisms. Notably, the UV-C band contributes 78% of the inactivation efficacy, but its photon penetration depth is limited by Mie scattering—the effective depth in solid matrices is only 200 ± 50 μm [50]. This results in the survival of pathogens in microbial shelter areas formed by vegetable surface folds, reaching up to 300% survival in smooth areas. To overcome this penetration bottleneck, a multiaxis dynamic irradiation system has been proposed. This system, by optimizing pulse angles and frequencies, improves the uniformity of shadow area treatment by 40% [49], providing key technical support for the inactivation of complex-structured vegetable ingredients such as cabbage and lettuce. Moreover, recent reports have shown that PLSs have a broad inactivation range, covering bacteria, yeasts, and molds [51] (Figure 4). Under its triple synergistic mechanism, it can inhibit or even kill Gram-negative bacteria (e.g., *Salmonella enterica*, *Pseudomonas aeruginosa*, and *Escherichia coli*), Gram-positive bacteria (e.g., *Listeria monocytogenes*, *Staphylococcus aureus*, *Bacillus subtilis*, and *Bacillus cereus*), yeast (e.g., *Saccharomyces cerevisiae*), and molds (e.g., *Penicillium citrinum* and *Aspergillus niger*).

### 3.3. Catalytic Infrared Radiation

Catalytic infrared radiation (CIR) is an innovative thermal inactivation technology based on multiphase catalytic oxidation (Figure 3). It generates infrared radiation with a characteristic wavelength of 3.3–3.8 μm through a surface combustion reaction mediated by a Pt-Pd bimetallic catalyst using a propane-air mixture [52,53]. Compared with conventional thermal sterilization, CIR offers two advantages. First, catalytic combustion increases the heat conversion efficiency to 65–75%, achieving 40–60% energy savings compared with resistive heating [54]. Secondly, the reaction byproducts are solely H_2_O and CO_2_, meeting clean production standards [55]. CIR exerts its microbial inactivation through a quadruple synergistic path, including the induction of irreversible conformational changes in proteins (denaturation enthalpy change ΔHm ≥ 150 kJ/mol), melting of DNA double helix structures, membrane phospholipid phase transition leading to increased permeability, and cleavage of β-1,4-glycosidic bonds in the peptidoglycan layer [50]. Owing to structural differences in microbial cell walls, the effects of these four CIR paths inevitably vary, ultimately resulting in the selective inactivation of microorganisms. Current research reports indicate that this technology can effectively kill bacteria such as *Staphylococcus aureus* and *Escherichia coli*, as well as *Aspergillus niger* [56,57] (Figure 4).

Moreover, notable selective antibacterial efficacy may also stem from the water gradient targeting effect, where the moisture content of microbial cells (generally >80%) is significantly greater than that of dried vegetable ingredients (≈10%), leading to selective enrichment of infrared energy within microbial bodies. This characteristic has been fully verified in the treatment of dried shiitake mushrooms [58]. When the water activity is 0.75, *Aspergillus niger* spores achieve 5.8 log inactivation due to their high water content, while the polysaccharide retention rate of the shiitake mushroom matrix remains >95%, the total phenol loss rate is <8%, and the surface microstructure integrity is well maintained (roughness change ΔRa < 0.2 μm).

### 3.4. Microwave

Microwave sterilization technology is based on the principle of electromagnetic energy transfer. Its core mechanism lies in the synergistic effect of thermal and nonthermal effects (Figure 3). When materials are placed in a microwave field (frequency range of 300 MHz–300 GHz), polar molecules undergo dipole polarization under the action of an alternating electric field, with molecular orientations aligning with the field strength direction at a frequency of billions of times per second. The intense friction generated between molecules leads to rapid and uniform heating within the material [59]. Research confirms that the thermal effect of microwave sterilization primarily stems from two aspects: the dielectric loss heat generated by the high-frequency vibration of polar molecules [60,61] and the Joule heating effect formed by ion conduction. This is the traditionally recognized sterilization principle of microwaves.

However, nonthermal sterilization brings new technological avenues for food material sterilization. Canumir et al. [62] revealed this special biological effect of microwave electromagnetic fields through *Escherichia coli* inactivation experiments. When the treatment power was increased from 720 W to 900 W, the D value of *E. coli* did not significantly change (*p* > 0.05), whereas the center temperature of apple juice exceeded the lethal threshold of 72 °C. Gong et al. [40] further validated this phenomenon. During the 915 MHz microwave treatment, although the surface temperature of the tomato juice (62.3 ± 1.2 °C) was lower than the center temperature (73.5 ± 0.8 °C), the surface microbial inactivation rate reached 1.8 times that of the center region (*p* < 0.05). This suggests that, in addition to the thermal effect, microwave fields may alter cell membrane potential distribution (ΔΨ≈15 mV) and ion channel permeability, interfering with the normal metabolic pathways of microorganisms. This nonthermal effect mechanism also leads to differential killing effects among microbial groups with different cell wall and membrane structures, resulting in selective inactivation. Furthermore, it is generally believed that microwave inactivation is the result of a synergistic effect between thermal and nonthermal effects, with the thermal effect contributing approximately 75–85%. However, under specific frequency bands (2.45 GHz) and field strength conditions (>10 kV/m), the nonthermal effect can significantly enhance inactivation efficacy. Under this synergistic effect of thermal and nonthermal effects, microwave sterilization can target difficult-to-eliminate *Bacillus*, *Clostridium*, *Penicillium*, and *Aspergillus strains* (Figure 4).

### 3.5. Radio Frequency

Radio frequency inactivation technology (3 kHz–300 MHz), a novel physical antimicrobial method (Figure 3), has significant advantages over traditional heat conduction methods in terms of its thermal dynamic characteristics [63]. Through the dual mechanisms of dipole rotation and ion migration, the radio frequency field enables the formation of a three-dimensional uniform thermal field within the material [64], increasing the thermal efficiency by 40–60%. Experiments conducted by Zhong and Sandeep [65] and Zhong et al. [66] using a radio frequency processing system (40.68 MHz, 30 kW) demonstrated that for Newtonian fluids (tap water), the time required to heat from 25 °C to 95 °C was only one-third of that needed for traditional heating, with an axial temperature coefficient of variation (CV) less than 1.5%. When non-Newtonian fluids (1% CMC solution) were processed, a unique temperature gradient field formed within the tubular reactor (centre wall ΔT ≈ 20 °C). This dynamic thermal distribution characteristic, combined with spatial differences in fluid residence time (center zone τ = 18 s, near-wall zone τ = 32 s), resulted in temperature uniformity of ±1.5 °C at the outlet. Compared with microwave technology, radio frequency sterilization has three core advantages in industrial applications. First, the electromagnetic wave penetration depth is increased by 2–3 orders of magnitude, allowing for the processing of materials with a maximum cross-section of 0.5 m^3^. Second, the energy conversion efficiency reaches 85–92%, which is approximately 15% higher than that of microwave systems. Third, continuous processing can be achieved through a multistage resonant cavity design, with a single machine processing capacity of 8–12 t/h. Zhang et al. [64] used 27.12 MHz radio frequency (RF) heating to treat walnut shells inoculated with *Staphylococcus aureus* ATCC 25923 and reported that RF heating exerts its sterilizing effect by disrupting cell membrane homeostasis, inhibiting cell wall synthesis, and interfering with energy metabolism. Currently, RF technology is mainly applied to the genera *Bacillus* and *Staphylococcus*, which are Gram-positive bacteria, as well as *Enterobacteriaceae* bacteria, which are Gram-negative bacteria (Figure 4).

## 4. Natural Essential Oil

Chemical preservatives, as crucial means of inactivation in the realm of food safety, are confronted with a significant challenge: they are primarily effective against free-floating microorganisms but remain incapable of eradicating pathogens firmly colonized on the surfaces of vegetable raw materials without compromising the sensory qualities of the product [67]. Amid growing consumer concerns regarding the potential toxicological risks and ecological impacts of synthetic preservatives, the development of green and sustainable natural antimicrobial systems has emerged as a focal point in food science research [68]. Plant essential oils, which are rich in a variety of bioactive volatile secondary metabolites, have garnered considerable attention because of their broad-spectrum antimicrobial and antioxidant activities [69] (Table 1).

Plant essential oils exhibit selective bactericidal effects on microbial communities adhering to vegetable raw materials, primarily through mechanisms involving membrane disruption, metabolic interference, and genetic material perturbation. First, membrane disruption serves as the primary pathway through which most essential oils exert their antimicrobial effects. For example, terpenes and phenols can penetrate microbial cell membranes owing to their lipophilic nature, leading to membrane depolarization and leakage of their intracellular contents [70]. Components such as camphor and β-caryophyllene in sage essential oils induce ATP efflux by altering membrane permeability, thereby achieving bacteriostatic effects [71]. Second, metabolic interference also plays a pivotal role. Eugenol and other constituents of clove essential oil can effectively inhibit extracellular polysaccharide synthesis, reduce bacterial motility, and disrupt biofilm structural integrity [72], offering novel insights into overcoming traditional antimicrobial resistance associated with biofilms. Furthermore, certain essential oil components can block genetic information transmission by embedding within the DNA double helix or inhibiting DNA repair enzyme activity. For example, the stable complex formed between eugenol and DNA significantly inhibits bacterial proliferation [73].

**Table 1:** Application of chemical inactivation technology based on natural essential oils in vegetable raw materials.

Name	Major Active Components	Mode of Action	Applications and Antibacterial Concentration	References
Clove Essential Oil	Eugenol, Acetyl Eugenol	Induces cytoplasmic aggregation; Disrupts cell wall and membrane systems; alters DNA structure	Cucumber, 2.5 mg/mL; Tomato paste 500 ppm;	[74,75]
Thyme Essential Oil	Thymol, p-Cymene, γ-Terpinene	Disrupts cell wall and membrane systems; Interferes with genetic material synthesis and metabolism	Iceberg Lettuce, Spinach 0.2% (*v*/*v)*;	[76,77]
Cinnamon Essential Oil	Trans-Cinnamaldehyde	Disrupts cell wall and membrane systems; Inhibits cellular energy metabolism	Lettuce, 5 μL/mL; Cucumber 0.1% (*v*/*v*);	[78,79]
Peppermint Essential Oil	Menthol, Menthone	Disrupts cell wall and membrane systems; Interferes with genetic material synthesis and metabolism	Corn Kernels, Peanut Kernels, 0.343 μL/mL;	[80]
Sage Essential Oil	Camphor, β-Caryophyllene, 1,8-Cineole	Disrupts cell wall and membrane systems	Potato, 0.22–0.75 μg/mL;	[81,82]
Rosemary Essential Oil	α-Pinene, 1,8-Cineole, Camphor	Disrupts cell wall and membrane systems	Spinach, Red Cabbage, Broccoli, 0.48 mg/mL;	[83,84]
Basil Essential Oil	Eugenol, Caryophyllene	Disrupts cell wall and membrane systems; inhibits cellular energy metabolism	Kohlrabi, 500 ppm;	[85,86]
Oregano Essential Oil	Carvacrol, Thymol	Disrupts cell wall and membrane systems	Lettuce, Beetroot, 0.6 μL/mL;	[87,88]

With respect to inactivation measures for fermented vegetable raw materials, the application of plant essential oils has demonstrated immense potential. Various studies have reported that diverse essential oils, through different active components and mechanisms, can inhibit and kill harmful microorganisms residing on the surfaces of vegetables (Figure 4). For example, clove essential oils, via eugenol and acetyl eugenol, can inhibit and eliminate *Escherichia coli* and *Aspergillus flavus* on cucumber [74] and tomato surfaces [75]. Thyme essential oils, through thymol, p-cymene, and γ-terpinene, can inhibit and eradicate *Bacillus cereus*, *Escherichia coli*, *Listeria monocytogenes*, and even *Aspergillus fumigatus* on lettuce and spinach [76]. Cinnamon essential oil, by means of trans-cinnamaldehyde, can inhibit and kill *Salmonella enterica* and *Fusarium solani* on lettuce [78] and cucumber [79]. Notably, despite the diverse modes of action of these essential oils, they share a common mechanism: the disruption of the cell wall and membrane systems of harmful microorganisms. Consequently, different microbial communities, due to variations in their cell wall and membrane structures, exhibit differential responses to essential oil inactivation, resulting in selective sterilization effects. However, the practical application of plant essential oils still faces significant challenges, particularly regarding regulatory approval for use in food systems. While many essential oils are generally recognized as safe (GRAS) for use as flavorings, their approval as antimicrobial preservatives faces stricter regulatory scrutiny [89,90]. Firstly, the concentrations required for effective antimicrobial action (Minimum Inhibitory Concentration—MIC) are often significantly higher than those used for flavoring [91,92]. Comprehensive toxicological data (e.g., genotoxicity and chronic exposure studies) at these higher doses are frequently lacking for many oils, which is a prerequisite for regulatory approval as a food additive preservative. Secondly, effective antimicrobial doses often exceed sensory thresholds, altering the product′s taste and aroma [16,93]. This creates a significant hurdle for approval, as regulators must balance efficacy with minimal impact on the sensory qualities of the food matrix. Additionally, their high volatility, photothermal sensitivity, and poor water solubility limit their processing stability and bioavailability [94,95,96,97]. To address these issues, researchers are focusing on synergistic enhancement techniques involving physical fields, with the five physical processing technologies described earlier serving as potential enhancement methods.

## 5. LAB Metabolite

In fermented vegetable systems, the succession patterns of microbial communities exhibit distinct stage-specific characteristics, with their metabolic activities directly determining the sensory quality, texture, and biosafety of the product [98]. Corruption bacteria (e.g., *Pseudomonas* and *Enterobacteriaceae*) and pathogens (e.g., *Staphylococcus aureus* and *Listeria monocytogenes*) on the surface of raw materials can easily trigger vegetable spoilage, bloating, mold growth, and toxin accumulation [99]. LAB secrete two core antimicrobial metabolites, organic acids and bacteriocins [100,101,102], to selectively inhibit and kill these harmful microorganisms (Figure 3), thereby ensuring biosafety while preserving the indigenous beneficial fermentative microbial community of the raw materials, which is crucial for protecting the regional flavor of fermented vegetables.

### 5.1. Organic Acids: Synergistic Antibacterial Effects of Constructing an Acidic Barrier

The organic acids produced by LAB are among the primary antimicrobial active products, including lactic acid, acetic acid, citric acid, and phenyllactic acid, among others. Through synergistic and competitive actions, organic acids can appropriately increase food sourness, lower the pH of the food environment to inhibit bacterial corruption, disrupt microbial membrane permeability, interfere with intracellular metabolic processes and hinder the growth and reproduction of various acid-intolerant pathogens, thereby prolonging the shelf life and safety of fermented vegetables [103,104]. The lactic acid, acetic acid, and propionic acid produced by LAB constitute an acidic antibacterial barrier, with mechanisms including proton gradient disruption and energy competition, membrane integrity damage, and metabolic enzyme inhibition. Proton gradient disruption and energy competition occur when organic acids dissociate into acid radical ions (ROO^−^) and protons (H^+^) within cells. The dissociated protons cannot cross the membrane via free diffusion, leading to an overload of intracellular H^+^ [105]. To maintain the cellular pH balance, bacteria actively transport hydrogen ions out of the cell, a process that consumes substantial ATP and energy, resulting in osmotic bactericidal effects [106]. Arrioja et al. [107] demonstrated that the cell-free supernatant derived from *Lactobacillus plantarum* NRRL B-4496, which contains approximately 2.06% lactic acid and 0.11% acetic acid, can reduce the load of Salmonella Typhimurium by 3.74 log_10_ CFU/g when used as a marinade for beef. Membrane integrity damage involves small-molecule organic acids penetrating the periplasmic space between the outer and inner cell membranes, where lipopolysaccharides and phospholipid groups are protonated, promoting the dissociation of outer membrane protein components and lipopolysaccharides, thereby destroying membrane integrity and causing leakage of the intracellular contents to achieve antibacterial effects [108]. Studies have shown that while H^+^ is released into the extracellular environment, K^+^ is pumped into the cell, disrupting the bacterial transmembrane proton motive force and increasing the intracellular osmotic pressure, leading to bacterial membrane rupture and content leakage, thus achieving bactericidal effects [109]. Metabolic enzyme inhibition refers to organic acids inactivating key enzymes (decarboxylases, dehydrogenases, catalases, etc.) in bacterial metabolic pathways, impeding normal bacterial metabolism. Research indicates that low concentrations of organic acids (acetic acid, citric acid, and lactic acid) interfere with energy metabolism by inhibiting key enzymes in the TCA cycle and glycolysis pathways of *Salmonella* [109]. Simultaneously, organic acids reduce the concentrations of metabolic products such as ATP and NAD, inhibiting the activity of enzymes related to DNA replication (e.g., DNA polymerase) [108]. Guimarães et al. [110] demonstrated that the organic acids produced by *Lactobacillus buchneri* UTAD104 and *Lactobacillus plantarum* UM55 inhibited the production of ochratoxin A more strongly than the inhibition of mycelial growth, suggesting that these organic acids may interfere with key enzymes in the ochratoxin A biosynthesis pathway. Phenyllactic acid, produced by LAB, has different antibacterial mechanisms than the aforementioned organic acids do [111]. Xu et al. [98] reported that phenyllactic acid, which disrupts bacterial motility and extracellular polysaccharide (EPS) biosynthesis, has significant antibiofilm activity against *Enterococcus faecalis*. A concentration of 10.0 mg/mL phenyllactic acid effectively removed 72 h mature biofilms on polystyrene and stainless-steel substrates, indicating its potential as a biofilm control agent.

### 5.2. Bacteriocins: Targeting Membrane Structures and Intracellular Metabolism

Bacteriocins are a class of antimicrobial peptides produced by certain bacteria during metabolism via ribosomal synthesis mechanisms that exhibit inhibitory effects on Gram-positive bacteria and food-borne fungi (Figure 4). The bacteriocins produced by LAB can inhibit various bacteria and pathogens, such as *Bacillus cereus*, yeast, *Staphylococcus aureus*, and *Penicillium, in pickled vegetables*. Ge et al. [112] isolated the strain *Lactobacillus paracasei* HD1–7 from Chinese pickle juice, which produces the bacteriocin HD1-7 capable of inhibiting multiple bacteria, bacteria, and *Saccharomyces cerevisiae*. Moon et al. [113] compared three different LAB strains and reported that vegetables fermented with *Lactobacillus citreum* GR1 presented increased bacteriocin activity, increased sensory quality and longer shelf-life. The antimicrobial mechanisms of LAB bacteriocins involve two primary molecular pathways: membrane integrity and permeability disruption and intracellular targeting (Table 2).

(1) Membrane integrity and permeability disruption: Most bacteriocins disrupt bacterial membrane integrity and permeability, forming transmembrane pores. In bacteria, the cell wall consists of a layer of peptidoglycan formed by negatively charged teichoic acids and N-acetylmuramic acid. Lipopolysaccharides, a vital component of the Gram-negative bacterial outer membrane, also carry a negative charge. LAB bacteriocins are generally cationic peptides with strong hydrophobicity and high isoelectric points, allowing them to bind to bacterial cell membranes. Studies have shown that the effectiveness of bacteriocins in targeting bacterial cell membranes and exerting antimicrobial activity is strongly influenced by their positive charge and interaction with cell membranes [114]. The bacteriocin Nisin, produced by Lactococcus lactis, is applied in dairy and canned foods at nanomolar concentrations to inhibit spoilage and pathogenic Gram-positive bacteria such as *Listeria monocytogenes*, *Staphylococcus aureus*, and *Clostridium botulinum* [115]. One of the antimicrobial mechanisms of Nisin is its ability to target the bacterial cell membrane, forming pores that lead to the leakage of intracellular components such as K^+^ and ATP, disrupting the membrane potential and ultimately impeding cell growth, thereby inhibiting *Staphylococcus Koch* [116]. The second antimicrobial mechanism of Nisin involves binding to the pyrophosphate group of Lipid II (a crucial precursor for peptidoglycan synthesis in bacterial cell walls), preventing cell wall synthesis and forming transmembrane pores, resulting in cell death [117].
(2) Intracellular targeting: The intracellular action of bacteriocins refers to their interference with normal cellular metabolic activities by binding to cell membrane surface receptors or accumulating within bacterial cells. This can be classified into several types. First, LAB bacteriocins can interfere with bacterial cell growth by inhibiting nucleotide and protein synthesis. Second, they can inhibit bacterial cell wall biosynthesis, such as by disrupting the production and degradation of precursor substances such as Lipid II, thereby obstructing bacterial proliferation and metabolism. Moreover, some LAB bacteriocins, such as RNA polymerase or DNA gyrase, can affect key enzyme activities within bacterial cells [118]. Additionally, certain LAB-derived bacteriocins can inhibit bacterial spore germination and proliferation through nonmembrane-targeting mechanisms. For example, they can nonspecifically degrade bacterial DNA or specifically cleave 16S rRNA, blocking the biosynthesis of related functional proteins [119]. In summary, the antibacterially active substance bacteriocin produced by LAB causes bacterial lysis and death by acting on cell membranes and intracellular substances, effectively inhibiting and killing pathogens and damaging bacteria during fermentation of fermented vegetables while preserving the LAB community, which plays a decisive role in quality and flavor formation.

However, the practical application of bacteriocins also faces significant challenges related to regulatory approval for use in food systems. Beyond nisin, the regulatory pathway for new bacteriocins is complex. Gaining approval requires a substantial body of evidence demonstrating not only efficacy but also safety through rigorous toxicological studies, which is a costly and time-consuming process [120,121]. Additionally, the regulatory status of bacteriocins varies globally. For instance, nisin is approved in the EU and the US, but other bacteriocins like pediocin may have approval in some countries but not others [122,123]. This lack of harmonization complicates their application in international food products. Therefore, while the scientific foundation for using bacteriocins is strong, translating this potential into widespread industrial application necessitates overcoming these significant regulatory hurdles by generating robust data on standardized efficacy, safety, and technological applicability.

## 6. Conclusions and Future Perspectives

In conclusion, the existing antimicrobial methods discussed in this paper, including cold plasma, electromagnetic waves, natural essential oils, and LAB metabolites, despite their diverse mechanisms, can achieve selective inactivation effects on different types of microbial communities in vegetable raw materials through fine-tuning of parameters, substrate concentrations, and action times. Through meticulously designed experimental protocols, the precise differentiation and eradication of harmful microbial communities while preserving beneficial fermentative microorganisms such as LAB will have profound implications for the processing of fermented vegetables. This not only adds extra value to the preservation of fermented vegetable raw materials by ensuring anticorrosion and freshness while retaining the indigenous microbial communities of raw material origin but also holds immeasurable significance for the production and processing of fermented vegetables with strong regional flavors. Compared with current methods that investigate regional flavor sources and directly add fixed strains or even genetically engineered LAB to mimic regional quality and flavor, the methods proposed in this paper are more cost-effective and feasible. Furthermore, tailoring exclusive sterilization measure combinations for different types of fermented vegetables, fully leveraging the characteristics and advantages of various sterilization technologies against specific microbial types, is expected to maximize the effects of selective sterilization. This not only opens new avenues for optimizing the quality and flavor of fermented vegetables but also provides important references for technological innovation and sustainable development in the fermented food industry.

In the future, with the continuous innovation and optimization of existing antimicrobial technologies, selective inactivation strategies for fermented vegetable raw materials will have broader development prospects. On the one hand, interdisciplinary integration (such as the intersection of physics, chemistry, biology, and food engineering) will drive the precision and intelligence of inactivation technologies, achieving more efficient, low-energy, and residue-free selective antimicrobial effects. For example, combining artificial intelligence with big data analysis can precisely predict and optimize inactivation parameters, customizing inactivation schemes for different microbial groups and fermented vegetable raw material characteristics. On the other hand, green and sustainable natural antimicrobial systems will become a research hotspot. The development and application of biobased antimicrobial agents such as plant essential oils and LAB metabolites are expected to reduce their environmental impact while ensuring food safety and meeting consumers’ demands for healthy and eco-friendly food. Moreover, the exploration and application of combined strategies will also be an important future direction, leveraging the synergistic effects of physical, chemical, and biological methods to achieve multidimensional and multilayered selective antimicrobial actions, further enhancing the quality and flavor of fermented vegetables. In summary, selective antimicrobial strategies based on existing antimicrobial technologies will inject new vitality into the transformation, upgrading, and sustainable development of the fermented vegetable industry.

## Figures and Tables

**Table 2:** Classes and properties of bacteriocins from LAB.

Class	Example	Characteristics of Bacteriocins
I	Ia: Lantibiotics (Nisin J)	Thermally stable, Thermostable
	Ib: Cyclic bacteriocins (Labyrinthopeptin A1)	
II	IIa: Pediocin-like bacteriocins (Acidocin A)	Non-lanthionine-containing thermostable small peptides
	IIb: Two-component or multicomponent bacteriocins (Plantaricin EF)	Two-peptide bacteriocin requiring synergistic action of both peptides for antimicrobial activity
	IIc: Leaderless circular bacteriocins (Gassericin A)	Characterized by covalently linked N- and C-terminal cyclic structures; exhibits enhanced stability under high temperatures, acidic conditions, and digestive enzymes compared to other Class II bacteriocins
	IId: Other linear non-pediocin-like single-component bacteriocins (Lacticin Z)	Unmodified, linear, and non-pediocin-like
III	IIIa: PG Hydrolase Bacteriocins (Zoocin A)	Bind to surface receptors of target cells, inducing cell lysis
	IIIb: Large Nonenzymatic Bacteriocins (Helveticin 34.9)	Disrupt cell membranes; heat-sensitive large proteins produced predominantly by Lactobacillus

## Data Availability

No new data were created or analyzed in this study. Data sharing is not applicable to this article.
